# Quantitative proteome analysis of Merkel cell carcinoma cell lines using SILAC

**DOI:** 10.1186/s12014-019-9263-z

**Published:** 2019-12-19

**Authors:** Ulana Kotowski, Boban M. Erović, Julia Schnöll, Victoria Stanek, Stefan Janik, Martin Steurer, Goran Mitulović

**Affiliations:** 1 Department of Otorhinolaryngology, Head and Neck Surgery, Medical University of Vienna, 1090 Vienna, Austria; 2 Institute of Head and Neck Diseases, Evangelical Hospital Vienna, 1180 Vienna, Austria; 3 Proteomics Core Facility, Medical University of Vienna, 1090 Vienna, Austria; 4 Clinical Department of Laboratory Medicine, Medical University of Vienna, Waehringer Guertel 18-20, 1090 Vienna, Austria

**Keywords:** Merkel cell carcinoma, Proteomics, SILAC, Quantitative, Histone variants

## Abstract

**Background:**

Merkel cell carcinoma (MCC) is an aggressive neuroendocrine tumour of the skin with growing incidence. To better understand the biology of this malignant disease, immortalized cell lines are used in research for in vitro experiments. However, a comprehensive quantitative proteome analysis of these cell lines has not been performed so far.

**Methods:**

Stable isotope labelling by amino acids in cell culture (SILAC) was applied to six MCC cell lines (BroLi, MKL-1, MKL-2, PeTa, WaGa, and MCC13). Following tryptic digest of labelled proteins, peptides were analysed by mass spectrometry. Proteome patterns of MCC cell lines were compared to the proteome profile of an immortalized keratinocyte cell line (HaCaT).

**Results:**

In total, 142 proteins were upregulated and 43 proteins were downregulated. Altered proteins included mitoferrin-1, histone H2A type 1-H, protein-arginine deiminase type-6, heterogeneous nuclear ribonucleoproteins A2/B1, protein SLX4IP and clathrin light chain B. Furthermore, several proteins of the histone family and their variants were highly abundant in MCC cell lines.

**Conclusions:**

The results of this study present a new protein map of MCC and provide deeper insights in the biology of MCC. Data are available via ProteomeXchange with identifier PXD008181.

## Background

Merkel cell carcinoma (MCC) is a rare malignant tumour of the skin with neuroendocrine differentiation [1, 2] and growing incidence rates ranging from 2 to 4 cases per million per year in Europe and the US, to 8 cases per million per year in Australia [3]. MCC shows a very aggressive behaviour with significant potential to build metastases and a high locoregional recurrence rate [4, 5]. The overall 10-year survival is reported to be 57.3% [6]. The main risk factors are UV radiation as MCC mainly appears in sun-exposed areas, and immunosuppression since the incidence is higher in HIV-infected patients, transplant recipients and patients with chronic lymphocytic leukaemia [7]. A further factor that plays an important role in the development of MCC is the Merkel cell polyomavirus [8]. Firstly described by Feng et al. in 2008 it can be found in up to 80% of the cases [9, 10]. However other biological processes are likely to be involved in the development of MCC.

A genomic profiling study of patients with MCC revealed that the most frequent abnormalities are related to the TP53 gene and the cell cycle pathway. Further abnormalities were found in the PI3K/AKT/mTOR pathway and DNA repair genes [11]. However, despite of changes at the chromosomal level, the dysfunction of biochemical pathways is expressed at the protein level. Therefore this proteomic study was conducted to gain deeper insights into the biology of MCC and possibly to find new molecular targets for therapy. Stable isotope labelling by amino acids in cell culture (SILAC) was applied to investigate the expression patterns of six MCC cell lines.

## Methods

### Cell culture

The human Merkel cell carcinoma cell lines BroLi, MKL-1, MKL-2, PeTa, WaGa, and MCC13 were a kind gift of Prof. Houben [12]. HaCaT, a human skin keratinocyte cell line was obtained from AddexBio (San Diego, CA, USA). Cells were cultured in RPMI buffer (Thermo Fisher Scientific, Waltham, MA, USA) supplemented with 10% fetal bovine serum (Thermo Fisher Scientific, Waltham, MA, USA) at 37 °C and 5% CO_2_ in a humidified incubator. For SILAC labelling cells were grown in SILAC Media supplemented either with ^13^C_6_ l-Lysine-2HCl (heavy) and l-Arginine-HCl (light) or with l-Lysine-2HCl (light) and l-Arginine-HCl (light) (Thermo Fisher Scientific, Waltham, MA, USA). Cells were cultured for at least ten cell doublings. Three biological replicates were cultured per each cell line, and these were merged into one sample upon cell lysis for further treatment. The labelling efficiency was estimated using the method described by Rappsilber et al. [13]. Briefly, incorporation efficiency of the heavy labeled amino acids into proteins was assessed in a pilot experiment, where a small aliquot of cells was lysed, and proteins were reduced, alkylated, and tryptically digested. The resulting peptides were subjected to MS analysis as described below. Heavy label incorporation into proteins obtained from cells was assessed to be more than 95%.

### Sample preparation and protein identification, quantification and analysis

#### Cell lysis and protein digests

MCC cells were lysed using the “Chemicon^®^-Total Protein Extraction Kit” containing TM buffer (HEPES, pH7.9, MgCl2, KCl, EDTA, Sucrose, Glycerol, Sodium deoxycholate, NP-40, Sodium Ortho Vanadate, Merck Millipore Vienna, Austria) according to manufacturer’s manual. Protein precipitation was performed using modified Wessel–Fluegge method as described elsewhere [14–17]. Lysed cell content was treated with methanol and dichloromethane and the interphase was collected. Proteins were pelleted by addition of methanol, air dried and dissolved in 50 mM triethylammonium bicarbonate (Sigma-Aldrich, Vienna, Austria). Protein content was measured using the Direct Detect FT-IR spectrophotometer (Merck Millipore Vienna, Austria) and equimolar amounts of proteins, 1 µg total proteins, from control and the MCC cells were mixed and submitted for tryptic digestion. Protein digest was performed using sequencing grade trypsin (Promega, Mannheim, Germany) as described earlier [17]. Briefly, proteins were reduced with 5 mM DTT for 30 min at 60 °C, and alkylated for 30 min with 15 mM iodoacetamide in the dark. Finally, porcine trypsin was added in a ratio 1:50 (w/w). After 16 h of incubation at 37 °C, aliquots of 20 µl were prepared and stored in 0.5 ml protein low-bind vials at − 20 °C until further usage. Iodoacetamide and DTT were purchased from Sigma-Aldrich (Vienna, Austria). All extractions and digestion steps were performed in protein low-bind vials of different volumes (Eppendorf, Vienna, Austria).

#### Nano-HPLC separation and MS detection

Methanol, was purchased from Merck (Vienna, Austria), 98% formic acid, acetonitrile, trifluoroacetic acid were purchased from Sigma-Aldrich (Vienna, Austria). HPLC grade water was prepared using an in-house Milli-Q plus device from Millipore (Vienna, Austria), and trifluoroethanol was purchased from Alfa Aesar (Karlsruhe, Germany). Reverse phase separation of tryptic peptides was conducted on a nanoRSLC UltiMate3000 (Thermo Fischer Scientific, Vienna, Austria) HPLC system consisting of an autosampler, thermal compartment and pumping module. PepMap C18 trap-column (300 µm ID × 5 mm length, 5 µm particle size, 100 Å pore size, Thermo Fisher Scientific, Vienna, Austria) was used for sample loading and desalting. The analytical column used for peptide separation was a 75 µm ID × 50 cm length Acclaim^®^ PepMap100 (C18, 3 µm particle size, 100 Å pore size, Thermo Fisher Scientific, Vienna, Austria). Both columns were operated at 60 °C in the column compartment as described earlier [15]. A total of 500 ng digested protein was injected onto the trap column at 30 µl/min and was loaded using the loading pump flow rate of 30 µl/min. After 10 min the switching valve changed the position and the trap column was switched into the flow path of the nano pump. Peptides were eluted from the trap column onto the separation column using the gradient described in Supplementary information and in Table 1. HPLC was hyphenated with the maXis Impact Q-Time-of-Flight mass spectrometer (Bruker, Bremen, Germany) equipped with a nano ESI captive spray source. Peptides were ionized using positive electrospray and Data-Dependent collision-induced-dissociation was used for peptide fragmentation (MS/MS data). MS data was acquired using the data-dependent mode with positive ionization. Capillary was set to 1.8 kV and 20 most intense ions were fragmented using the collisional induced fragmentation by ramping the collisional energy from 15 to 35 eV. Fragmented ions were excluded from further fragmentation for 60 s.

**Table 1:** An overview of upregulated proteins identified in Merkel cell carcinoma cell lines

Cell line	UniProt entry name	Protein name	Gene symbol	Ratio (H/L)
BroLi	MFRN1_HUMAN	Mitoferrin-1	SLC25A37	9.64
	SF01_HUMAN	Splicing factor 1	SF1	1.92
	N4BP1_HUMAN	NEDD4-binding protein 1	N4BP1	1.65
MKL-1	H2A1H_HUMAN	Histone H2A type 1-H	HIST1H2AH	5.25
	HMGN2_HUMAN	Non-histone chromosomal protein HMG-17	HMGN2	3.86
	C2C2L_HUMAN	C2 domain-containing protein 2-like	C2CD2L	3.63
	DPY30_HUMAN	Protein dpy-30 homolog	DPY30	3.56
	STON1_HUMAN	Stonin-1	STON1	3.18
	PREX2_HUMAN	Phosphatidylinositol 3,4,5-trisphosphate-dependent Rac exchanger 2 protein	PREX2	3.11
	CCAR1_HUMAN	Cell division cycle and apoptosis regulator protein 1	CCAR1	2.96
	RFC1_HUMAN	Replication factor C subunit 1	RFC1	2.89
	ATPB_HUMAN	ATP synthase subunit beta, mitochondrial	ATP5B	2.87
	ROAA_HUMAN	Heterogeneous nuclear ribonucleoprotein A/B	HNRNPAB	2.64
	DJB14_HUMAN	DnaJ homolog subfamily B member 14	DNAJB14	2.53
	SF3B1_HUMAN	Splicing factor 3B subunit 1	SF3B1	2.47
	RL29_HUMAN	60S ribosomal protein L29	RPL29	2.44
	HNRPM_HUMAN	Heterogeneous nuclear ribonucleoprotein M	HNRNPM	2.39
	SUMO4_HUMAN	Small ubiquitin-related modifier 4	SUMO4	2.38
	NDUS7_HUMAN	NADH dehydrogenase [ubiquinone] iron-sulfur protein 7, mitochondrial	NDUFS7	2.29
	HSP7C_HUMAN	Heat shock cognate 71 kDa protein	HSPA8	2.19
	CH60_HUMAN	60 kDa heat shock protein, mitochondrial	HSPD1	2.10
	BSDC1_HUMAN	BSD domain-containing protein 1	BSDC1	2.06
	RS30_HUMAN	40S ribosomal protein S30	FAU	2.01
	MXRA5_HUMAN	Matrix-remodeling-associated protein 5	MXRA5	1.96
	ROA2_HUMAN	Heterogeneous nuclear ribonucleoproteins A2/B1	HNRNPA2B1	1.89
	ROA1_HUMAN	Heterogeneous nuclear ribonucleoprotein A1	HNRNPA1	1.89
	ROA3_HUMAN	Heterogeneous nuclear ribonucleoprotein A3	HNRNPA3	1.89
	NPM_HUMAN	Nucleophosmin	NPM1	1.80
	CH10_HUMAN	10 kDa heat shock protein, mitochondrial	HSPE1	1.70
	STK24_HUMAN	Serine/threonine-protein kinase 24	STK24	1.70
	HNRPC_HUMAN	Heterogeneous nuclear ribonucleoproteins C1/C2	HNRNPC	1.66
	H2B1O_HUMAN	Histone H2B type 1-O	HIST1H2BO	1.60
	PABP1_HUMAN	Polyadenylate-binding protein 1	PABPC1	1.58
	I23O2_HUMAN	Indoleamine 2,3-dioxygenase 2	IDO2	1.54
MKL-2	PADI6_HUMAN	Protein-arginine deiminase type-6	PADI6 PAD6	9.47
	CTL2_HUMAN	Choline transporter-like protein 2	SLC44A2	6.74
	SRSF3_HUMAN	Serine/arginine-rich splicing factor 3	SRSF3	5.87
	HMGN2_HUMAN	Non-histone chromosomal protein HMG-17	HMGN2	5.10
	PABP1_HUMAN	Polyadenylate-binding protein 1	PABPC1	4.02
	NDUS7_HUMAN	NADH dehydrogenase [ubiquinone] iron-sulfur protein 7, mitochondrial	NDUFS7	3.84
	H2B1H_HUMAN	Histone H2B type 1-H	HIST1H2BH	3.15
	COF1_HUMAN	Cofilin-1	CFL1	2.30
	LRRT3_HUMAN	Leucine-rich repeat transmembrane neuronal protein 3	LRRTM3	2.27
	CH10_HUMAN	10 kDa heat shock protein, mitochondrial	HSPE1	2.20
	ACM2_HUMAN	Muscarinic acetylcholine receptor M2	CHRM2	2.14
	S38AA_HUMAN	Putative sodium-coupled neutral amino acid transporter 10	SLC38A10	2.14
	ROA1_HUMAN	Heterogeneous nuclear ribonucleoprotein A1	HNRNPA1	2.08
	WDR62_HUMAN	WD repeat-containing protein 62	WDR62	2.02
	ROA2_HUMAN	Heterogeneous nuclear ribonucleoproteins A2/B1	HNRNPA2B1	2.01
	STMN1_HUMAN	Stathmin	STMN1	1.80
	TM266_HUMAN	Transmembrane protein 266	TMEM266	1.77
	H2B2E_HUMAN	Histone H2B type 2-E	HIST2H2BE	1.66
	TBA1A_HUMAN	Tubulin alpha-1A chain	TUBA1A	1.64
	TBA1B_HUMAN	Tubulin alpha-1B chain	TUBA1B	1.64
	HNRPK_HUMAN	Heterogeneous nuclear ribonucleoprotein K	HNRNPK	1.60
	PRDX2_HUMAN	Peroxiredoxin-2	PRDX2	1.50
PeTa	ROA2_HUMAN	Heterogeneous nuclear ribonucleoproteins A2/B1	HNRNPA2B1	4.46
	ROA3_HUMAN	Heterogeneous nuclear ribonucleoprotein A3	HNRNPA3	4.46
	H2A1B_HUMAN	Histone H2A type 1-B/E	HIST1H2AB	4.23
	H2AJ_HUMAN	Histone H2A.J	H2AFJ	4.23
	ROA1_HUMAN	Heterogeneous nuclear ribonucleoprotein A1	HNRNPA1	2.98
	NIBL1_HUMAN	Niban-like protein 1	FAM129B	2.97
	CTL2_HUMAN	Choline transporter-like protein 2	SLC44A2	2.96
	REPS2_HUMAN	RalBP1-associated Eps domain-containing protein 2	REPS2	2.72
	URGCP_HUMAN	Up-regulator of cell proliferation	URGCP	2.69
	H14_HUMAN	Histone H1.4	HIST1H1E	2.67
	H33_HUMAN	Histone H3.3	H3F3A	2.39
	K0895_HUMAN	Uncharacterized protein KIAA0895	KIAA0895	2.36
	H15_HUMAN	Histone H1.5	HIST1H1B	2.17
	H2B1N_HUMAN	Histone H2B type 1-N	HIST1H2BN	1.91
	H2B2E_HUMAN	Histone H2B type 2-E	HIST2H2BE	1.90
	PRDX2_HUMAN	Peroxiredoxin-2	PRDX2	1.80
WaGa	SLX4I_HUMAN	Protein SLX4IP	SLX4IP	33.84
	TMM56_HUMAN	Transmembrane protein 56	TMEM56	8.88
	S38AA_HUMAN	Putative sodium-coupled neutral amino acid transporter 10	SLC38A10	6.38
	COQ6_HUMAN	Ubiquinone biosynthesis monooxygenase COQ6, mitochondrial	COQ6	4.57
	NEK5_HUMAN	Serine/threonine-protein kinase Nek5	NEK5	4.36
	PEX16_HUMAN	Peroxisomal membrane protein PEX16	PEX16	4.33
	PEAK1_HUMAN	Pseudopodium-enriched atypical kinase 1	PEAK1	3.95
	PCBP2_HUMAN	Poly(rC)-binding protein 2	PCBP2	3.83
	HS71A_HUMAN	Heat shock 70 kDa protein 1A	HSPA1A	3.66
	FBX41_HUMAN	F-box only protein 41	FBXO41	3.59
	HNRPC_HUMAN	Heterogeneous nuclear ribonucleoproteins C1/C2	HNRNPC	3.55
	HMMR_HUMAN	Hyaluronan mediated motility receptor	HMMR	3.37
	SMYD5_HUMAN	SET and MYND domain-containing protein 5	SMYD5	3.27
	MBNL2_HUMAN	Muscleblind-like protein 2	MBNL2	2.47
	H4_HUMAN	Histone H4	HIST1H4A	2.45
	HNF4A_HUMAN	Hepatocyte nuclear factor 4-alpha	HNF4A	2.35
	ASPG_HUMAN	*N*(4)-(beta-*N*-acetylglucosaminyl)-l-asparaginase	AGA	2.20
	H2B1O_HUMAN	Histone H2B type 1-O	HIST1H2BO	1.99
	H2B1D_HUMAN	Histone H2B type 1-D	HIST1H2BD	1.97
	TBB5_HUMAN	Tubulin beta chain	TUBB	1.89
	UGGG1_HUMAN	UDP-glucose:glycoprotein glucosyltransferase 1	UGGT1	1.80
	UB2J1_HUMAN	Ubiquitin-conjugating enzyme E2 J1	UBE2J1	1.80
	RLA2_HUMAN	60S acidic ribosomal protein P2	RPLP2	1.77
	SF01_HUMAN	Splicing factor 1	SF1	1.71
	HNRL2_HUMAN	Heterogeneous nuclear ribonucleoprotein U-like protein 2	HNRNPUL2	1.71
	ROAA_HUMAN	Heterogeneous nuclear ribonucleoprotein A/B	HNRNPAB	1.70
	POP1_HUMAN	Ribonucleases P/MRP protein subunit POP1	POP1	1.69
	PRDX2_HUMAN	Peroxiredoxin-2	PRDX2	1.68
	CE57L_HUMAN	Centrosomal protein CEP57L1	CEP57L1	1.67
	CCDC6_HUMAN	Coiled-coil domain-containing protein 6	CCDC6	1.67
	KCTD9_HUMAN	BTB/POZ domain-containing protein KCTD9	KCTD9	1.66
	ADCY1_HUMAN	Adenylate cyclase type 1	ADCY1	1.59
	S35E2_HUMAN	Solute carrier family 35 member E2	SLC35E2	1.55
	ATP5J_HUMAN	ATP synthase-coupling factor 6, mitochondrial	ATP5J	1.51
	ATPA_HUMAN	ATP synthase subunit alpha, mitochondrial	ATP5A1	1.50
MCC13	CLCB_HUMAN	Clathrin light chain B	CLTB	4.71
	HSPB1_HUMAN	Heat shock protein beta-1	HSPB1	3.23
	SNX4_HUMAN	Sorting nexin-4	SNX4	2.83
	RL23A_HUMAN	60S ribosomal protein L23a	RPL23A	2.82
	PROF1_HUMAN	Profilin-1	PFN1	2.51
	H2B1C_HUMAN	Histone H2B type 1-C/E/F/G/I	HIST1H2BC	2.47
	ZN407_HUMAN	Zinc finger protein 407	ZNF407	2.44
	H2B1K_HUMAN	Histone H2B type 1-K	HIST1H2BK	2.42
	OPRD_HUMAN	Delta-type opioid receptor	OPRD1	2.42
	HGB1A_HUMAN	Putative high mobility group protein B1-like 1	HMGB1P1	2.35
	PSME1_HUMAN	Proteasome activator complex subunit 1	PSME1	2.29
	PRDX1_HUMAN	Peroxiredoxin-1	PRDX1	2.22
	CAC1G_HUMAN	Voltage-dependent T-type calcium channel subunit alpha-1G	CACNA1G	2.20
	H2B2F_HUMAN	Histone H2B type 2-F	HIST2H2BF	2.18
	K2C5_HUMAN	Keratin, type II cytoskeletal 5	KRT5	2.17
	RL4_HUMAN	60S ribosomal protein L4	RPL4	2.15
	LDHB_HUMAN	l-Lactate dehydrogenase B chain	LDHB	2.14
	MT1E_HUMAN	Metallothionein-1E	MT1E	2.14
	ILRL2_HUMAN	Interleukin-1 receptor-like 2	IL1RL2	1.94
	MYPC2_HUMAN	Myosin-binding protein C, fast-type	MYBPC2	1.93
	NONO_HUMAN	Non-POU domain-containing octamer-binding protein	NONO	1.84
	LMNA_HUMAN	Prelamin-A/C	LMNA	1.82
	K2C8_HUMAN	Keratin, type II cytoskeletal 8	KRT8	1.66
	NUCL_HUMAN	Nucleolin	NCL	1.64
	PDLI1_HUMAN	PDZ and LIM domain protein 1	PDLIM1	1.64
	RLA1_HUMAN	60S acidic ribosomal protein P1	RPLP1	1.64
	BAF_HUMAN	Barrier-to-autointegration factor	BANF1	1.63
	NPM_HUMAN	Nucleophosmin	NPM1	1.61
	HNRPK_HUMAN	Heterogeneous nuclear ribonucleoprotein K	HNRNPK	1.61
	YBOX1_HUMAN	Nuclease-sensitive element-binding protein 1	YBX1	1.60
	H2AJ_HUMAN	Histone H2A.J	H2AFJ	1.56
	RL34_HUMAN	60S ribosomal protein L34	RPL34	1.55
	ROA1_HUMAN	Heterogeneous nuclear ribonucleoprotein A1	HNRNPA1	1.52
	RS28_HUMAN	40S ribosomal protein S28	RPS28	1.52
	CQ047_HUMAN	Uncharacterized protein C17orf47	C17orf47	1.52

All raw data were converted into Mascot “mgf” files by using Bruker’s Data Analysis and these files were searched against the Swissprot database (version of November 2016) of human proteins using ProteinScape V 3.1.5 474 (Bruker, Bremen, Germany) and Mascot V2.6 (Matrix Science, London, UK). Protein quantitation was performed using WARP-LC V1.3.136 (Bruker, Bremen, Germany).

All samples were analysed as technical triplicates to ensure statistical sound data and avoid artefacts due to variations in ionization efficiency.

Detailed information on separation gradient, the MS settings, and the data search and quantitation can be found in supplemental information (Additional file 1).

### Term enrichment analysis

Heavy/light ratios where calculated using WARP-LC v. 1.3 (Bruker, Bremen, Germany). Proteins in MCC cell lines with H/L ratios of > 1.5 or < 0.5 where considered as significantly differently abundant. To put differential protein abundance into biologic context, Cytoscape (Seattle, WA, USA) in combination with ClueGO/CluePedia (a Cytoscape plug-in) was used with default parameters except for following: Database Gene Ontology Biological Process, levels between 4 and 13 and GO Fusion set on true [18]. p-values where corrected for multitesting according to Benjamini Hochberg.

## Results and discussion

### Differentially expressed proteins in MCC cell lines and the control cell line

**Fig. 1:**
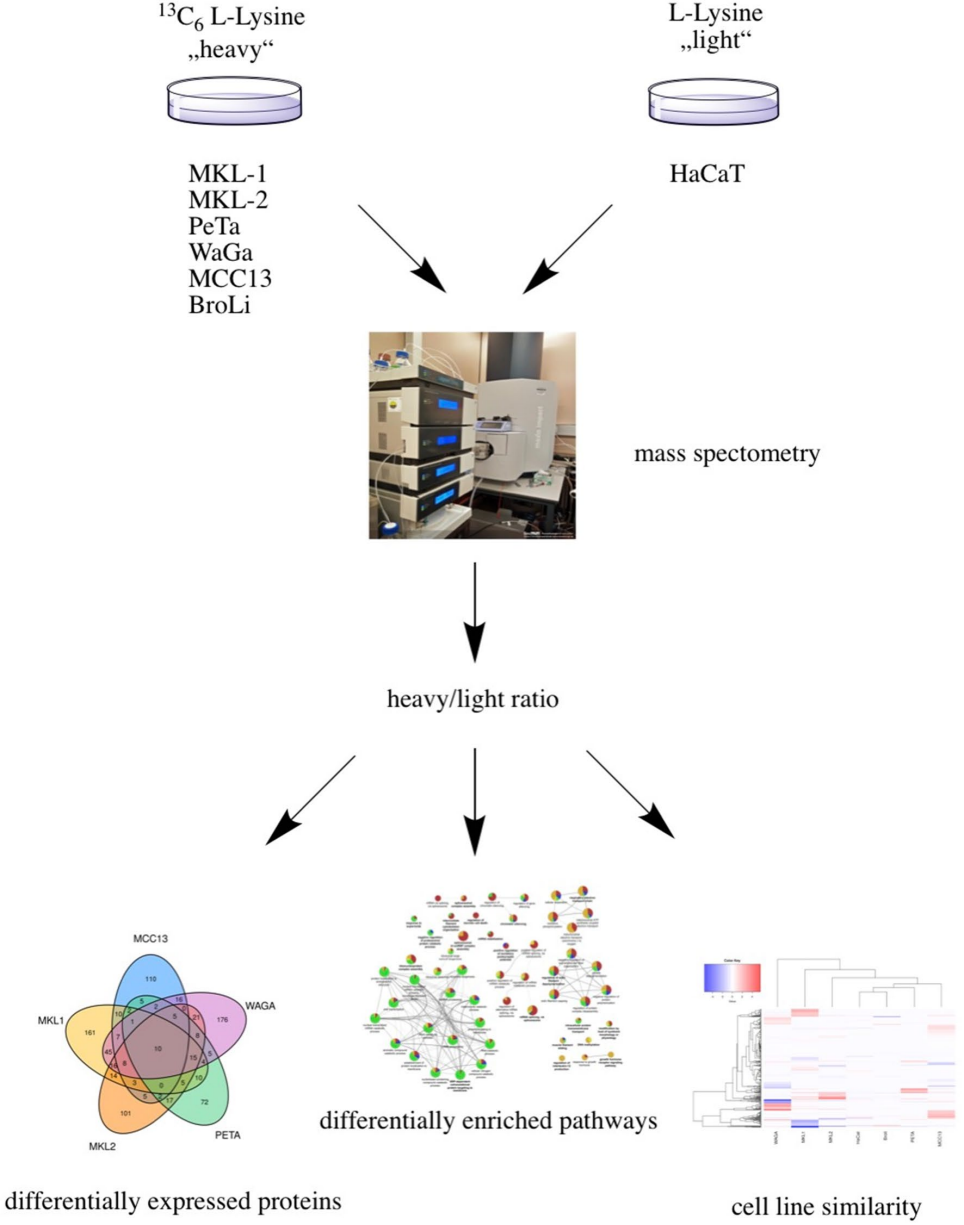
Experimental design of the study. MCC cells were cultured in medium supplemented with ^13^C_6_l-Lysine-2HCl (heavy) and HaCaT cells were cultured in medium supplemented with l-Lysine-2HCl (light). After tryptic digest of labelled proteins, peptides were analysed by mass spectrometry. A heat map was created to show cell line similarity. Specific proteins of each cell line (MCC13, MKL-1, MKL-2, PeTa and WaGa) were related in a Venn diagram. Furthermore, differentially enriched pathways were analysed. A heavy to light ratio of identified proteins was calculated and the up- and downregulation of MCC specific proteins was compared to the reference cell line HaCaT (Tables 1, 2)

The SILAC method was used in six MCC cell lines to determine quantitative changes of proteins at the proteome level. Proteins detected in MCC cells were compared to the reference cell line HaCaT (Fig. 1). We chose the keratinocyte cell line HaCaT as reference cell line since, to our best knowledge, there is no commercially or otherwise available cell line with healthy Merkel cells.

**Fig. 2:**
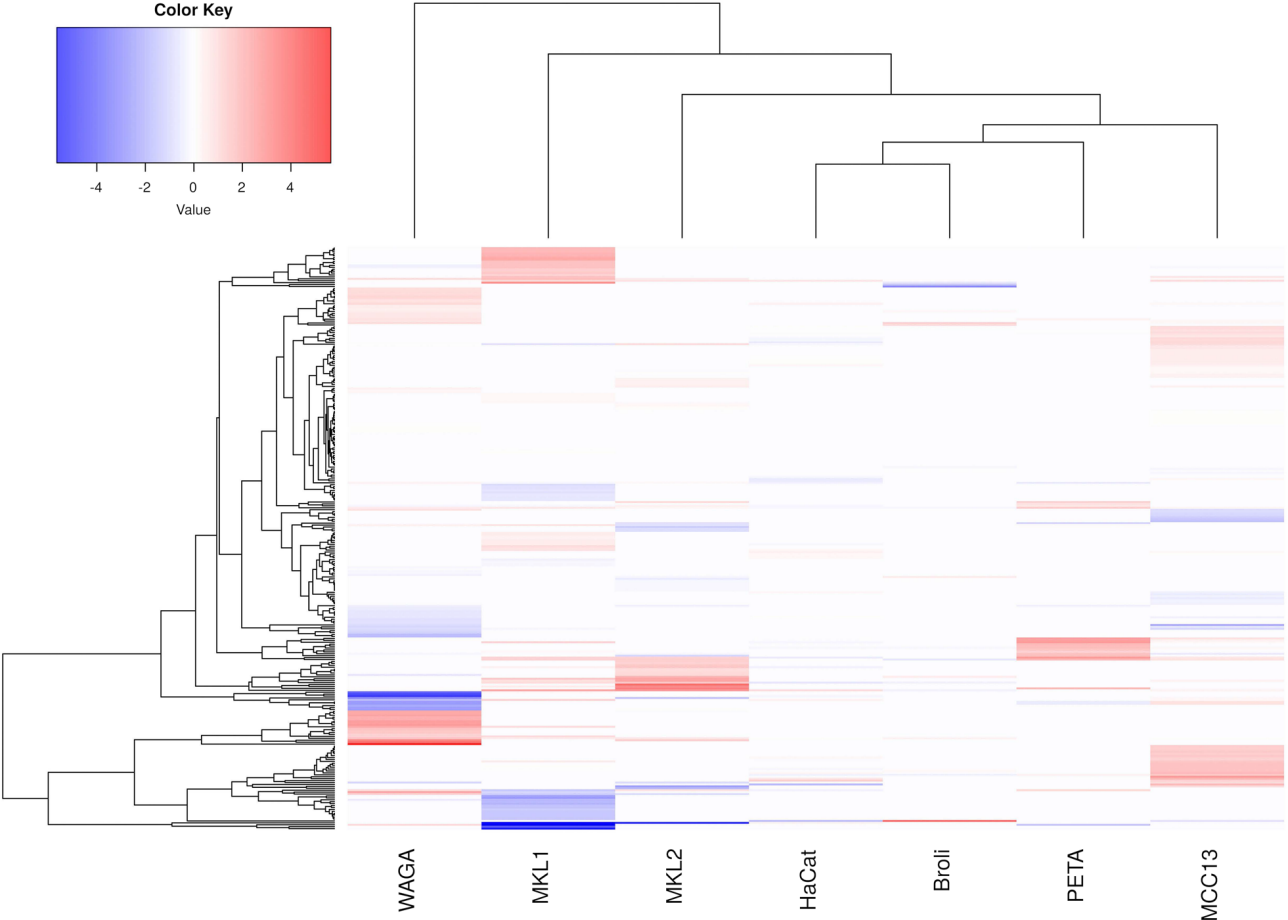
Analysis of cell line similarity. This figure displays the hierarchical clustering of cell lines based on the H/L ratio of differentially abundant proteins. The colour represents the z-normalized H/L ratio over all samples. Red = synthesis upregulated, blue = synthesis downregulated in comparison to HaCaT cells. Every cell line is characterized by a distinct pattern of specifically abundant proteins. Noteworthy, the BroLi is comparatively similar to HaCaT (the control), whereas WAGA displays considerable differences to the rest of the cell line

In order to visualize the similarity and the difference between the particular cell lines, a heat map was created showing all quantified proteins. A list of proteins (overlapping and specific) for each sample is provided as Additional file 2: Table S1. As seen in Fig. 2 every cell line has its own distinct protein abundance pattern. The most similar cell line compared to the control cell line HaCaT was BroLi, whereas WaGa differed significantly from the other cell lines. All cell lines originated from different old patients and different anatomic locations. While WaGa was derived from ascites of a 67 years old man, MKL-1 was derived from a nodal metastasis of a 26 years old man. MKL-2 stem from a 72 years old man and the localization is unknown. BroLi was obtained from pleural effusion of a 55 year old man [19]. MCC13 was gained from a nodal metastasis of a 80 year old female patient and is called in literature also “variant” MCC cell line since unlike BroLi, MKL-1, MKL-2, PeTa and WaGa, it is a Merkel cell polyomavirus negative cell line and lacks some typical markers in immunohistochemical staining [20]. Nevertheless we decided to include this cell line into our study since a number of studies in the field of MCC research are still performed using this particular cell line.

**Table 2:** An overview of downregulated proteins identified in Merkel cell carcinoma cell lines

Cell line	UniProt entry name	Protein name	Gene symbol	Ratio (H/L)
BroLi	H10_HUMAN	Histone H1.0	H1F0	0.45
	MGAP_HUMAN	MAX gene-associated protein	MGA	0.17
MKL-1	ASXL1_HUMAN	Putative Polycomb group protein ASXL1	ASXL1	0.48
	TBB5_HUMAN	Tubulin beta chain	TUBB TUBB5	0.48
	TAF2_HUMAN	Transcription initiation factor TFIID subunit 2	TAF2	0.45
	FBLN2_HUMAN	Fibulin-2	FBLN2	0.45
	PRDX2_HUMAN	Peroxiredoxin-2	PRDX2	0.44
	SEPT5_HUMAN	Septin-5	Sep-05	0.44
	FUS_HUMAN	RNA-binding protein FUS	FUS	0.44
	BICD2_HUMAN	Protein bicaudal D homolog 2	BICD2	0.43
	SRRT_HUMAN	Serrate RNA effector molecule homolog	SRRT	0.41
	MAK_HUMAN	Serine/threonine-protein kinase MAK	MAK	0.40
	CBX5_HUMAN	Chromobox protein homolog 5	CBX5	0.36
	CHSP1_HUMAN	Calcium-regulated heat-stable protein 1	CARHSP1	0.34
	NUCB2_HUMAN	Nucleobindin-2	NUCB2	0.31
	TPIS_HUMAN	Triosephosphate isomerase	TPI1	0.26
	TRI13_HUMAN	E3 ubiquitin-protein ligase TRIM13	TRIM13	0.22
	L37A3_HUMAN	Leucine-rich repeat-containing protein 37A3	LRRC37A3	0.08
	CMGA_HUMAN	Chromogranin-A	CHGA	0.03
	KCTD9_HUMAN	BTB/POZ domain-containing protein KCTD9	KCTD9	0.02
	1433Z_HUMAN	14-3-3 protein zeta/delta	YWHAZ	0.02
MKL-2	ALAT1_HUMAN	Alanine aminotransferase 1	GPT	0.49
	GRP75_HUMAN	Stress-70 protein, mitochondrial	HSPA9	0.40
	ULK2_HUMAN	Serine/threonine-protein kinase ULK2	ULK2	0.34
	NUCL_HUMAN	Nucleolin	NCL	0.24
	1433Z_HUMAN	14-3-3 protein zeta/delta	YWHAZ	0.04
PeTa	KCTD9_HUMAN	BTB/POZ domain-containing protein KCTD9	KCTD9	0.45
WaGa	TMX1_HUMAN	Thioredoxin-related transmembrane protein 1	TMX1	0.39
	RS30_HUMAN	40S ribosomal protein S30	FAU	0.38
	CPLX3_HUMAN	Complexin-3	CPLX	0.37
	CA052_HUMAN	UPF0690 protein C1orf52	C1orf52	0.27
	RSMB_HUMAN	Small nuclear ribonucleoprotein-associated proteins B and B’	SNRPB	0.23
	RS28_HUMAN	40S ribosomal protein S28	RPS28	0.22
	CQ047_HUMAN	Uncharacterized protein C17orf47	C17orf47	0.22
	G3P_HUMAN	Glyceraldehyde-3-phosphate dehydrogenase	GAPDH	0.21
	GRP75_HUMAN	Stress-70 protein, mitochondrial	HSPA9	0.17
	SAE2_HUMAN	SUMO-activating enzyme subunit 2	UBA2	0.09
	SRSF7_HUMAN	Serine/arginine-rich splicing factor 7	SRSF7	0.06
	MARCS_HUMAN	Myristoylated alanine-rich C-kinase substrate	MARCKS	0.05
MCC13	WNK4_HUMAN	Serine/threonine-protein kinase WNK4	WNK4	0.48
	HNRPU_HUMAN	Heterogeneous nuclear ribonucleoprotein U	HNRNPU	0.44
	RL18A_HUMAN	60S ribosomal protein L18a	RPL18A	0.42
	FUBP1_HUMAN	Far upstream element-binding protein 1	FUBP1	0.27

**Fig. 3:**
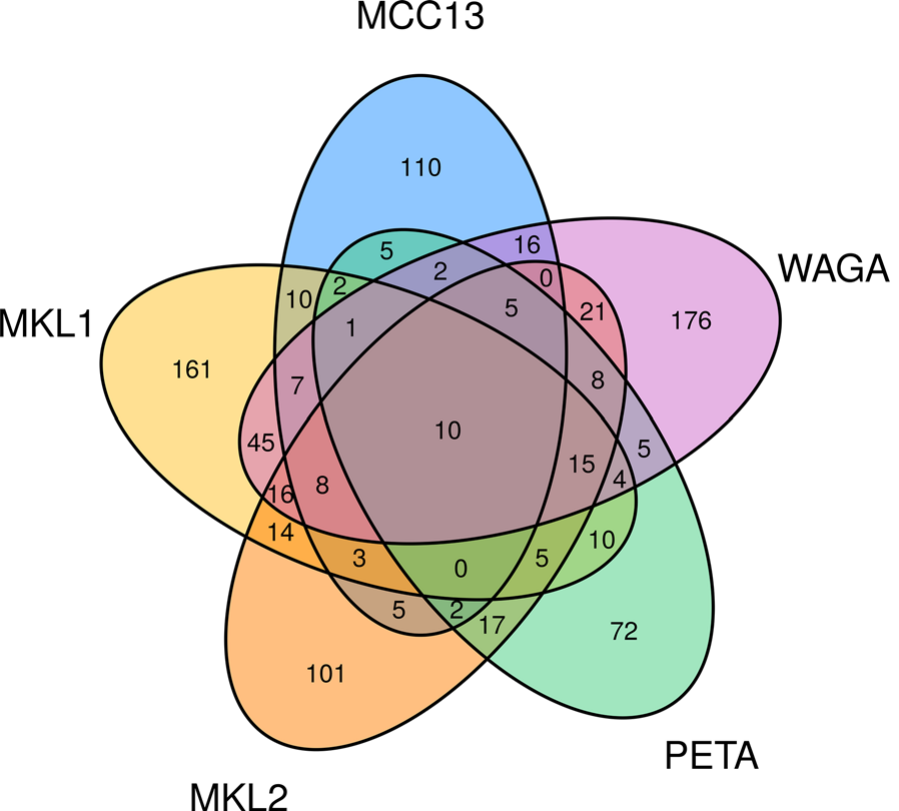
Specifically expressed proteins. This figure displays a Venn diagram of the specific proteins for the cell lines MKL-1, MKL-2, PeTa, WaGa, and MCC13. First, each cell line was compared to the reference cell line HaCaT and the proteins specific to each MCC cell line were determined. Due to the small difference between HaCaT and BroLi, BroLi has been omitted. Few proteins were found in several cell lines at the same time and only 10 proteins were found to be common in all cell lines. These 10 proteins are shown in the middle of the Venn diagram (alpha 2-HS glycoprotein, inter-alpha-trypsin inhibitor heavy chain 2, FUS RNA binding protein, mechanistic target of rapamycin, SUB1 homolog transcriptional regulator, Y-box binding protein 1, serine and arginine rich splicing factor 2, testis specific 10 interacting protein, sperm associated antigen 5 and heterogeneous nuclear ribonucleoprotein A/B). A complete list of all proteins identified is provided in Additional file 3: Table S2

Next, the protein profile of MCC cell lines was compared with the reference cell line HaCaT. Proteins present specifically in the MCC cells were determined. Then a Venn diagram was constructed. Since BroLi was the cell line with the least number of proteins and the difference between HaCaT and BroLi was small, the cell line BroLi has been omitted. Figure 3 shows a Venn diagram with the specific proteins for the cell lines MKL-1, MKL-2, PeTa, WaGa, and MCC13. Remarkably, only 10 proteins were found in all five cell lines at the same time: alpha 2-HS glycoprotein, inter-alpha-trypsin inhibitor heavy chain 2, FUS RNA binding protein, mechanistic target of rapamycin, SUB1 homolog transcriptional regulator, Y-box binding protein 1, serine and arginine rich splicing factor 2, testis specific 10 interacting protein, sperm associated antigen 5 and heterogeneous nuclear ribonucleoprotein A/B. A complete list of all specific proteins is provided as Additional file 3: Table S2.

### Term enrichment analysis of proteins

**Fig. 4:**
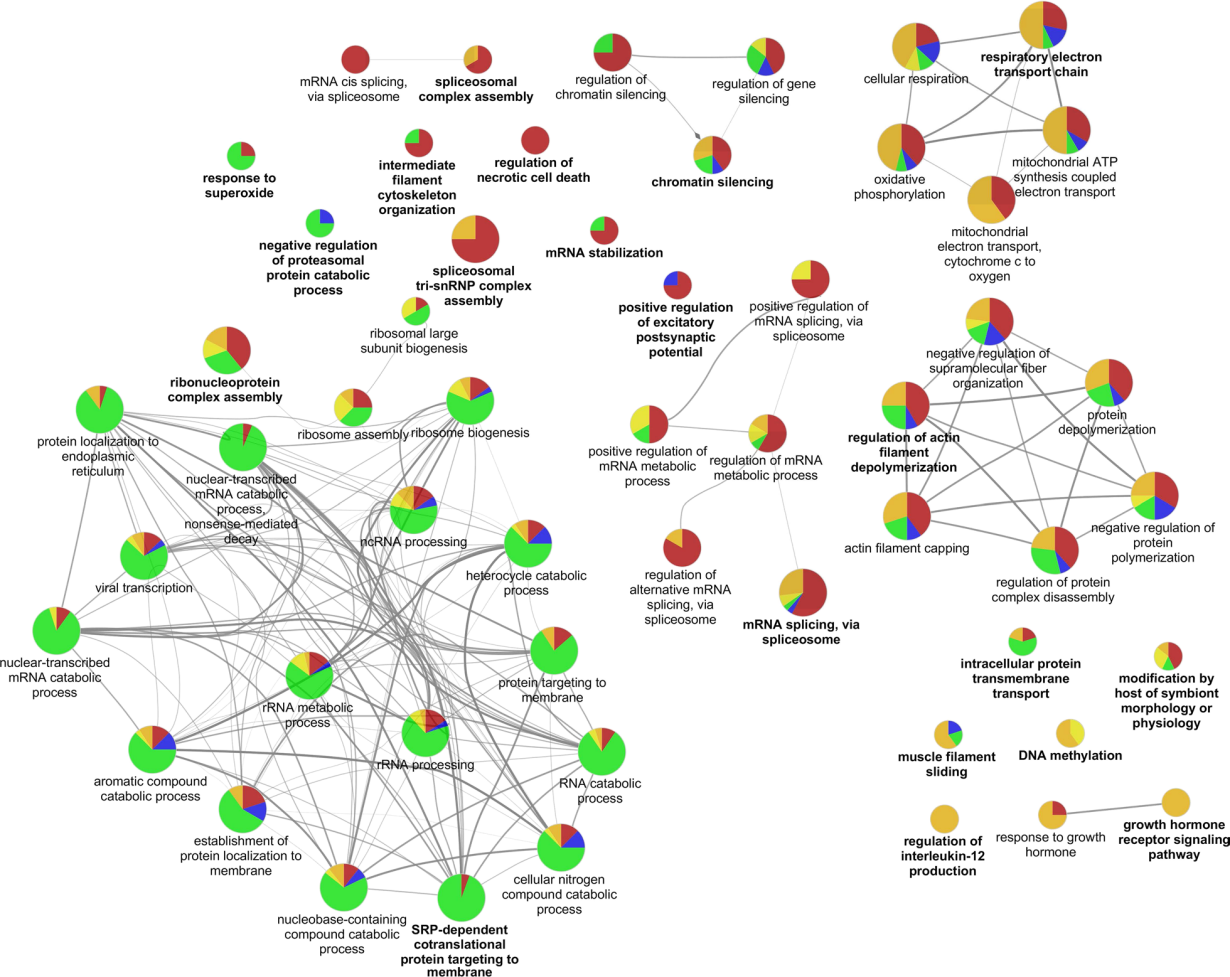
Enriched GO-Terms as detected by CLUEGO. Again, BroLi has been omitted due to the small differences between Broli and HaCaT. The diameter of the circles denotes the *p* value (corrected, Benjamini–Hochberg). The color denotes the percentage of proteins associated with the respective cell line. Multiple pathways were involved in different cell lines. Blue: MKL-1, green: MKL-2, yellow: PeTa, orange: WaGa, red: MCC13

It is of crucial importance and of highest interest to identify and quantify biological processes involved in the biology of cancer. Term Enrichment Analysis using ClueGO showed that multiple pathways where affected by differentially represented proteins (Fig. 4). Cellular processes like metabolic processes, protein folding, and signal transductions were affected. In particular, viral transcription was present in all cell lines but mostly in MKL-2. This can be explained by the fact that the Merkel cell polyomavirus has an important function in the pathogenesis of the development of MCC [21]. In the cell line MKL-2 also several mRNA and rRNA processes were more prevalent. In MCC13 the spliceosomal complex assembly was very active together with filament cytoskeleton organization and regulation of cell death. Further processes that play a role in cancer cell motility, like regulation of actin filament depolymerization [22] were enriched in several cell lines.

### Overexpression of multiple proteins in different MCC cell lines

In total, 317 dysregulated (i.e. up- and downregulated) proteins with significance threshold of p < 0.05 were identified. Proteins altered > 1.5-fold were considered as upregulated and proteins altered < 0.5-fold were considered as downregulated. Based on these criteria, 142 proteins were identified as upregulated and 43 proteins were downregulated. The differently abundant proteins and their ratios are shown in Tables 1 and 2.

Bioinformatic analysis revealed that different cell lines have individual protein profiles. None of the dysregulated proteins was present in all tested cell lines at the same time. However, a high occurrence of histone variants was detected in all cell lines except in BroLi. In more detail, only three upregulated and two downregulated proteins were identified in the BroLi cell line. BroLi cell line is a very slowly growing cell line with a doubling time of 5 days [19] and this could be the reason why only a limited number of proteins were identified.

For the BroLi cell line, mitoferrin-1 was found to be 9.64-fold upregulated compared to HaCaT cell line. Mitoferrin-1 is a protein involved in the mitochondrial iron transport and storage [23]. As iron is an important co-factor in DNA synthesis, dysregulated iron metabolism in cells is believed to play a role in tumorigenesis. The disturbance in iron transport between cytosol and mitochondrion is thought to lead to mitochondrial dysfunction and it therefore may contribute to tumour formation and propagation [24].

In MKL-2 cells, protein-arginine deiminase type-6 was upregulated 9.47-fold compared to the control cell line making it the most differently regulated protein for this cell line. This protein is an enzyme involved in post-translational modifications, which can have substantial effects on the structure and function of proteins. Citrullination is one such post-translational modification being catalysed by the family of protein arginine deiminase (PADs) enzymes. Five isoenzymes (PAD1-4 and 6) are known and they were identified in different types of tissue [25]. An overexpression of PADs has been detected in diseases like rheumatoid arthritis, neurologic diseases and cancer. In particular, the overexpression of PAD4 is associated with cancer since it plays a role in histone citrullination [26]. We identified PAD6, an isoenzyme mainly found in oocytes and embryos, to be the most abundant protein in the cell line MKL-2. Although the relation of PAD6 and cancer has not been described in the literature so far, we assume that it can be of interest due to its high occurrence.

Furthermore, we identified the heterogeneous nuclear ribonucleoprotein A2/B1 (hnRNPA2/B1) to be the most upregulated protein in the PeTa cell line. The hnRNPs are a group of proteins binding to RNA and playing a role in mRNA processing [27]. So far, hnRNPA2/B1 was found to be overexpressed in lung cancer were it promotes tumour growth by activation of COX-2 signalling [28, 29]. Furthermore, it was also found to be up regulated in hepatoma cell lines, gastric cancer, breast cancer and glioblastoma [30–32] but it was not described for Merkel cell carcinoma yet.

In the WaGa cell line, SLX4IP (SLX4 interacting protein) was the protein showing the highest upregulation. SLX4 is a DNA repair protein and it coordinates structure-specific endonucleases [33] but its role in cancer has not been determined up to now.

Finally, clathrin light chain B was the protein with the highest upregulation in the MCC13 cell line. Clathrin light chain B is a part of the clathrin protein, which is the main component of vesicles involved in intracellular transport. Recently, it was reported that clathrin light chains promote cell migration and therefore may play a role in cancer metastasis [34].

In the PeTa cell line various histones and their variants were found to be dysregulated compared to the control cell line HaCaT. Histones are substantial components for the packaging of the DNA in the chromosomes. The smallest packaging units are nucleosomes consisting of DNA wrapped around a histone octamer. A histone octamer in turn consists of two copies of each of the core histones: H2A, H2B, H3, and H4, being the smallest units. The linker histone H1 holds the nucleosome together and is the fifth member of the histone protein family [35]. Beside their structural function, histones play an important role in DNA replication and transcription regulation. Recently, it became evident that changes in histone expression are associated with cancer since an altered nucleosome structure can lead to instability and accessibility for different transcription factors [36]. So far, most of the histone variants were found in the histone H1, H2A, H2B, and H3 family. Some variants have been studied more detailed, but for many variants the function is still not known [37]. Furthermore, some histones serve as markers for cellular proliferation. In case of MCC, Henderson et al. used H3KT (histone-associated mitotic marker H3K79me3T80ph) and PHH3 (phosphohistone H3) as surrogates for detecting mitotic figures. Detection of H3KT and PHH3 correlated with a worse overall survival [38].

In the current study, proteins from all five major histone families with 15 different subfamily members were differently abundant in MCC cell lines compared to control samples. In particular, H2A1H and H2B1O were found to be overexpressed in MKL-1 and H2B1H and H2B2E were upregulated in MKL-2. Most of the highly abundant histone variants were found in the cell line PeTa: H2A1B, H2AJ, H1.4, H3.3, H1.5, H2B1N, and H2B2E. Furthermore, H2B1O and H2B1D were identified to be upregulated in WaGa, and H2B1C, H2B1K, H2B2F and H2AJ in MCC13. The role of histone variants in the development of carcinomas has been discussed and described in a number of publications [39–46]. As described in a recent review, canonical histones can be replaced with variant histones after environmental-stress-induced DNA damage repair, which subsequently results in a change in chromatin structure and stability [47]. A well-known environmental-stress factor is UV radiation, which in turn is a recognized risk factor for the development of Merkel cell carcinomas. This study shows for the first time that histone variants play an important role in the biology of Merkel cell carcinomas. A large number of histone variants was identified in all examined cell lines, except for the BroLi cell line.

Another interesting group of proteins that were identified as dyregulated in several MCC cell lines were the heat shock proteins (HSPs). It is a group of proteins that inhibit the unfolding or denaturation of cellular proteins and therefore being known as molecular chaperones whose expression is induced by stress. The major groups are classified according to their sizes and imply HSP10, HSP27, HSP40, HSP60, HSP70, and HSP90. Recent studies have shown that HSPs are highly expressed in many malignant tumours and due to their important role in cell proliferation and differentiation they are involved in carcinogenesis and metastasis [48, 49]. In case of MCC, presence of HSP70 seems to be necessary for the interaction of large T antigen and the tumour-suppressing retinoblastoma protein. In detail, the large T antigen is an oncoprotein expressed by polyomavirus affected cells and Merkel cell carcinoma in turn is highly associated with polyomavirus [9]. Binding of large T antigen to retinoblastoma protein leads to inactivation of retinoblastoma protein [50] and subsequently to cell proliferation via activation of cell cycle progression associated genes [51].

Beside HSP70, we found HSP60 and co-chaperone HSP10 to be overexpressed in the tested MCC cell lines. Actively produced by cancer cells, HSP60 exhibits a protective effect against cell stressors like chemotherapeutics. In particular, HSP60 stabilizes the anti-apoptotic protein survivin, a protein over-expressed in most human tumours, and therefore it inhibits apoptosis. Furthermore, HSP60 builds a complex with p53, which leads to the loss of the pro-apoptotic function of p53 and this process again results in inhibition of apoptosis [52].

## Conclusions

In conclusion, this work provides an additional insight in the biology of Merkel cell carcinoma. Multiple dysregulated proteins from various pathways were identified. The most abundant proteins were mitoferrin-1, histone H2A type 1-H, protein-arginine deiminase type-6, heterogeneous nuclear ribonucleoproteins A2/B1, protein SLX4IP and clathrin light chain B. Furthermore, the family of histone variants was frequently upregulated. In overall, each Merkel cell carcinoma cell line has its own distinct proteomic profile. This may be due to the biological heterogeneity of MCC. In this study we could demonstrate for the first time the similarities and differences between commonly used MCC cell lines.

Current analysis can be significantly improved by: (a) using multidimensional separation approach for fractionation of tryptic peptides and (b) using a more sensitive mass spectrometer. We are aware of this facts and new analysis of these samples are currently being processed. However, taking into consideration that these data are the very first describing differences of putative Merkel cells we are confident that they can provide valuable help for researchers addressing this condition.

## Supplementary information

**Additional file 1:** Additional methods information.

**Additional file 2: Table S1.** Shows overlapping and specific proteins for each cell line.

**Additional file 3. Table S2.** Lists all proteins that are shown in the Venn diagram (Fig. 3). First each cell line was compared to the reference cell line HaCaT. Then the cell line specific proteins of each cell line (MCC13, MKL-1, MKL-2, PeTa and WaGa) were related in a Venn diagram to show similarities and differences.

## Data Availability

The mass spectrometry proteomics data have been deposited to the ProteomeXchange Consortium via the PRIDE partner repository with the dataset identifier PXD008181 and 10.6019/pxd008181. Reviewer account details for the peer reviewing (to be deleted upon reviewing): Username: reviewer55818@ebi.ac.uk. Password: r6eVep0h.

## Abbreviations

MCC: Merkel cell carcinoma
SILAC: stable isotope labelling by amino acids in cell culture
PAD: protein arginine deiminase
hnRNP: heterogeneous nuclear ribonucleoprotein
SLX4IP: SLX4 interacting protein
HSP: heat shock protein
