# Supplementary material for: Quantitative proteome analysis of Merkel cell carcinoma cell lines using SILAC
Source: Clin Proteomics. 2019 Dec 19;16:42. doi: 10.1186/s12014-019-9263-z (PMC6921584; doi:10.1186/s12014-019-9263-z)
Supplement: Supplementary file 1 — Additional file 1: Additional methods information. [file 12014_2019_9263_MOESM1_ESM.docx]

**Additional Information on “Quantitative proteome analysis of Merkel cell carcinoma cell lines using SILAC”**

**HPLC Separation**

The nano HPLC Separation was performed using a nanoRSLC UltiMate 3000 HPLC system by Thermo Fisher.

Mobile phases applied for sample loading, desalting, and separation were:

- 0.1% aqueous TFA solution cooled to 3°C and delivered to the trap column in the column oven at 30µl/min
- Mobile phases for peptide separation on the nano separation column were:

A: 95% Acetonitrile, 5% Water, 0.1% Formic acid

B: 50% Acetonitrile, 30% Methanol, 10% 2,2,2-Trifluoroethanol, 10% Water, 0.1% Formic acid

- Autosampler loading solvent was 0.1% aqueous TFA
- Injector and trap column wash were performed with 100% 2,2,2-Trifluoroethanol

A cleaning run was performed between each sample in order to prevent carryover. The Chromelon v. 6.8 parameters are shown below as a text file. It can be copied and pasted in any Chromeleon v. 6.8, or earlier. For newer Chromeleon versions or different LC control systems this has to be adapted accordingly.

| Nano Separation Gradient |  |  |  |
| --- | --- | --- | --- |
|  |  |  |  |
| **Retention time (min)** | **Flow (µl/min)** | **%A** | **%B** |
| 0 | 0.30 | 95.00 | 5.00 |
| 10 | 0.30 | 95.00 | 5.00 |
| 100 | 0.30 | 90.00 | 10.00 |
| 200 | 0.30 | 75.00 | 25.00 |
| 275 | 0.30 | 60.00 | 40.00 |
| 285 | 0.30 | 40.00 | 60.00 |
| 290 | 0.30 | 10.00 | 90.00 |
| 295 | 0.30 | 10.00 | 90.00 |
| 296 | 0.30 | 95.00 | 5.00 |
| 300 | 0.30 | 95.00 | 5.00 |
|  |  |  |  |
|  |  |  |  |
| Column Valve Switching Times |  |  |  |
|  |  |  |  |
| **Retention time (min)** | **Valve A** | **Valve B** |  |
| 0 | 1_2 | 1_2 |  |
| 10 | 1_2 | 10_1 |  |
| 286 | 1_2 | 1_2 |  |
|  |  |  |  |

Mascot Search parameters used in ProteinScape are shown below:


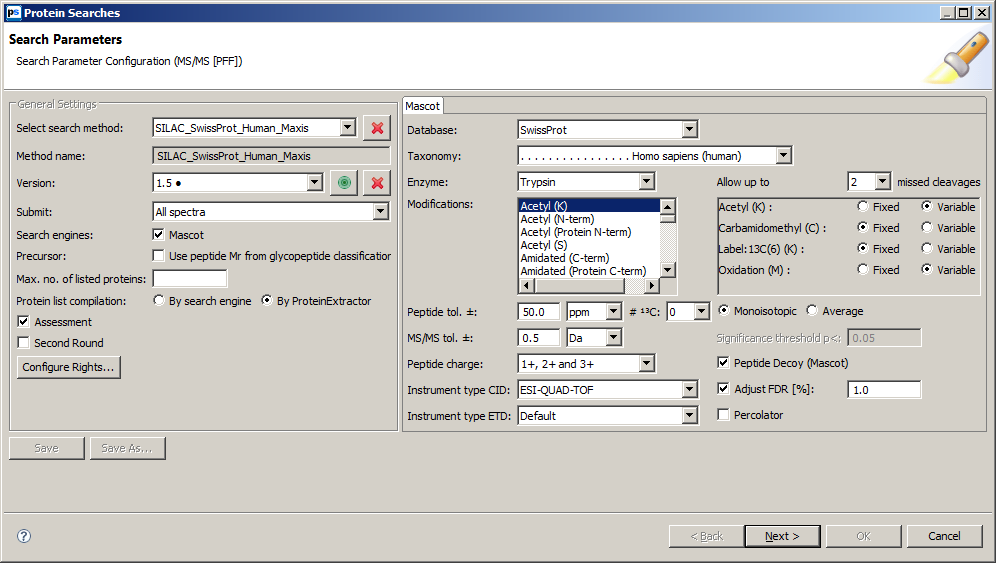


**HPLC Program**

Sampler.TempCtrl = On

Sampler.Temperature.Nominal = 5.0 [°C]

Sampler.Temperature.LowerLimit = 4.0 [°C]

Sampler.Temperature.UpperLimit = 45.0 [°C]

Sampler.ReadyTempDelta = None

ColumnOven.TempCtrl = On

ColumnOven.Temperature.Nominal = 60.0 [°C]

ColumnOven.Temperature.LowerLimit = 20.0 [°C]

ColumnOven.Temperature.UpperLimit = 75.0 [°C]

EquilibrationTime = 0.5 [min]

ColumnOven.ReadyTempDelta = 1.0 [°C]

LoadingPump.Pressure.LowerLimit = 0 [bar]

LoadingPump.Pressure.UpperLimit = 500 [bar]

LoadingPump.MaximumFlowRampDown = 10 [µl/min²]

LoadingPump.MaximumFlowRampUp = 10 [µl/min²]

LoadingPump.%A.Equate = "%A"

LoadingPump.%B.Equate = "%B"

%C.Equate = "%C"

NC_Pump.Pressure.LowerLimit = 0 [bar]

NC_Pump.Pressure.UpperLimit = 800 [bar]

NC_Pump.MaximumFlowRampDown = 0.500 [µl/min²]

NC_Pump.MaximumFlowRampUp = 0.500 [µl/min²]

NC_Pump.%A.Equate = "%A"

NC_Pump.%B.Equate = "%B"

DrawSpeed = 1000 [nl/s]

DrawDelay = 5000 [ms]

DispSpeed = 2000 [nl/s]

DispenseDelay = 2000 [ms]

WasteSpeed = 4000 [nl/s]

WashSpeed = 4000 [nl/s]

LoopWashFactor = 2.000

SampleHeight = 3.000 [mm]

PunctureDepth = 7.000 [mm]

WashVolume = 100.000 [µl]

RinseBetweenReinjections = No

LowDispersionMode = Off

InjectMode = UserProg

LoadingPump_Pressure.Step = 0.01 [s]

LoadingPump_Pressure.Average = On

NC_Pump_Pressure.Step = 0.01 [s]

NC_Pump_Pressure.Average = On

NC_Pump_Press_RightBlk.Step = 0.01 [s]

NC_Pump_Press_RightBlk.Average = On

NC_Pump_Press_LeftBlk.Step = 0.01 [s]

NC_Pump_Press_LeftBlk.Average = On

ColumnOven_Temp.Step = 0.01 [s]

ColumnOven_Temp.Average = Off

UV.Data_Collection_Rate = 2.0 [Hz]

TimeConstant = 1.20 [s]

UV_VIS_1.Wavelength = 214 [nm]

ValveRight = 1_2

ReagentAVial= R1

ReagentBVial= R2

Start_MS.State Off

ValveLeft = 1_2

PrepVial= B5

UdpInjectValve Position=Inject

UdpSyringeValve Position=Needle

UdpDraw From=ReagentAVial, Volume=15.000, SyringeSpeed=1000, SampleHeight=4.000

UdpMixWait Duration=2

UdpDraw From=ReagentAVial, Volume=0.000, SyringeSpeed=GlobalSpeed, SampleHeight=4.000

UdpInjectValve Position=Load

UdpDraw From=SampleVial, Volume=Volume, SyringeSpeed=GlobalSpeed, SampleHeight=4.000

UdpMixWait Duration=2

UdpDraw From=SampleVial, Volume=0.000, SyringeSpeed=GlobalSpeed, SampleHeight=4.000

UdpDraw From=ReagentAVial, Volume=15.000, SyringeSpeed=1000, SampleHeight=4.000

UdpMixWait Duration=2

UdpDraw From=ReagentAVial, Volume=0.000, SyringeSpeed=GlobalSpeed, SampleHeight=4.000

UdpInjectValve Position=Inject

UdpInjectMarker

UdpMixNeedleWash Volume=100.000

UdpMixWait Duration=800

UdpDraw From=ReagentBVial, Volume=25.000, SyringeSpeed=4000, SampleHeight=4.000

UdpMixWait Duration=2

UdpDispense To=Drain, Volume=10.000, SyringeSpeed=GlobalSpeed, SampleHeight=4.000

UdpInjectValve Position=Load

UdpDraw From=ReagentBVial, Volume=25.000, SyringeSpeed=4000, SampleHeight=4.000

UdpMixWait Duration=2

UdpDraw From=ReagentBVial, Volume=0.000, SyringeSpeed=GlobalSpeed, SampleHeight=4.000

UdpInjectValve Position=Inject

UdpMixWait Duration=300

UdpInjectValve Position=Load

UdpDraw From=ReagentAVial, Volume=25.000, SyringeSpeed=4000, SampleHeight=4.000

UdpMixWait Duration=2

UdpDraw From=ReagentAVial, Volume=0.000, SyringeSpeed=GlobalSpeed, SampleHeight=4.000

UdpInjectValve Position=Inject

UdpMixNeedleWash Volume=300.000

0.000 Autozero

NC_Pump.Flow = 0.300 [µl/min]

NC_Pump.%B = 5.0 [%]

LoadingPump.Flow = 20.000 [µl/min]

LoadingPump.%B = 0.0 [%]

%C = 0.0 [%]

Wait UV.Ready and LoadingPump.Ready and NC_Pump.Ready and ColumnOven.Ready and Sampler.Ready and PumpModule.Ready

Inject

LoadingPump_Pressure.AcqOn

NC_Pump_Pressure.AcqOn

NC_Pump_Press_RightBlk.AcqOn

NC_Pump_Press_LeftBlk.AcqOn

ColumnOven_Temp.AcqOn

UV_VIS_1.AcqOn

NC_Pump.Flow = 0.300 [µl/min]

LoadingPump.Flow = 20.000 [µl/min]

LoadingPump.%B = 0.0 [%]

%C = 0.0 [%]

NC_Pump.%B = 5.0 [%]

10.000 NC_Pump.%B = 5.0 [%]

ValveLeft = 1_2

ValveRight = 10_1

NC_Pump.Flow = 0.300 [µl/min]

15.000 Start_MS.State On

15.100 Start_MS.State Off

30.000 LoadingPump.Flow = 20.000 [µl/min]

LoadingPump.%B = 0.0 [%]

%C = 0.0 [%]

32.000 LoadingPump.Flow = 2.000 [µl/min]

LoadingPump.%B = 0.0 [%]

%C = 0.0 [%]

100.000 NC_Pump.%B = 10.0 [%]

NC_Pump.Flow = 0.300 [µl/min]

200.000 NC_Pump.%B = 25.0 [%]

NC_Pump.Flow = 0.300 [µl/min]

275.000 NC_Pump.%B = 40.0 [%]

NC_Pump.Flow = 0.300 [µl/min]

280.000 LoadingPump.Flow = 2.000 [µl/min]

LoadingPump.%B = 0.0 [%]

%C = 0.0 [%]

285.000 NC_Pump.%B = 60.0 [%]

NC_Pump.Flow = 0.300 [µl/min]

286.000 ValveLeft = 1_2

LoadingPump.Flow = 20.000 [µl/min]

LoadingPump.%B = 0.0 [%]

%C = 0.0 [%]

ValveRight = 1_2

290.000 NC_Pump.%B = 90.0 [%]

NC_Pump.Flow = 0.300 [µl/min]

295.000 NC_Pump.%B = 90.0 [%]

NC_Pump.Flow = 0.300 [µl/min]

296.000 NC_Pump.%B = 5.0 [%]

NC_Pump.Flow = 0.300 [µl/min]

300.000 NC_Pump.Flow = 0.300 [µl/min]

NC_Pump.%B = 5.0 [%]

LoadingPump_Pressure.AcqOff

NC_Pump_Pressure.AcqOff

NC_Pump_Press_RightBlk.AcqOff

NC_Pump_Press_LeftBlk.AcqOff

ColumnOven_Temp.AcqOff

UV_VIS_1.AcqOff

End

**HPLC Program used for column cleaning**

Sampler.TempCtrl = On

Sampler.Temperature.Nominal = 15.0 [°C]

Sampler.Temperature.LowerLimit = 4.0 [°C]

Sampler.Temperature.UpperLimit = 45.0 [°C]

Sampler.ReadyTempDelta = None

ColumnOven.TempCtrl = On

ColumnOven.Temperature.Nominal = 60.0 [°C]

ColumnOven.Temperature.LowerLimit = 20.0 [°C]

ColumnOven.Temperature.UpperLimit = 75.0 [°C]

EquilibrationTime = 0.5 [min]

ColumnOven.ReadyTempDelta = 1.0 [°C]

LoadingPump.Pressure.LowerLimit = 0 [bar]

LoadingPump.Pressure.UpperLimit = 500 [bar]

LoadingPump.MaximumFlowRampDown = 10 [µl/min²]

LoadingPump.MaximumFlowRampUp = 10 [µl/min²]

LoadingPump.%A.Equate = "%A"

LoadingPump.%B.Equate = "%B"

%C.Equate = "%C"

NC_Pump.Pressure.LowerLimit = 0 [bar]

NC_Pump.Pressure.UpperLimit = 800 [bar]

NC_Pump.MaximumFlowRampDown = 0.300 [µl/min²]

NC_Pump.MaximumFlowRampUp = 0.300 [µl/min²]

NC_Pump.%A.Equate = "%A"

NC_Pump.%B.Equate = "%B"

DrawSpeed = 1000 [nl/s]

DrawDelay = 5000 [ms]

DispSpeed = 20000 [nl/s]

DispenseDelay = 2000 [ms]

WasteSpeed = 40000 [nl/s]

WashSpeed = 40000 [nl/s]

LoopWashFactor = 2.000

SampleHeight = 3.000 [mm]

PunctureDepth = 7.000 [mm]

WashVolume = 300.000 [µl]

RinseBetweenReinjections = No

LowDispersionMode = Off

InjectMode = UserProg

LoadingPump_Pressure.Step = 0.01 [s]

LoadingPump_Pressure.Average = On

NC_Pump_Pressure.Step = 0.01 [s]

NC_Pump_Pressure.Average = On

NC_Pump_Press_RightBlk.Step = 0.01 [s]

NC_Pump_Press_RightBlk.Average = On

NC_Pump_Press_LeftBlk.Step = 0.01 [s]

NC_Pump_Press_LeftBlk.Average = On

ColumnOven_Temp.Step = 0.01 [s]

ColumnOven_Temp.Average = Off

UV.Data_Collection_Rate = 2.0 [Hz]

TimeConstant = 1.20 [s]

UV_VIS_1.Wavelength = 214 [nm]

LoadingPump.Flow = 5.000 [µl/min]

LoadingPump.%B = 0.0 [%]

%C = 0.0 [%]

LoadingPump.Curve = 5

ReagentAVial= R1

ReagentBVial= R2

Start_MS.State Off

ValveLeft = 10_1

PrepVial= B5

UdpInjectValve Position=Inject

UdpSyringeValve Position=Needle

UdpDraw From=ReagentBVial, Volume=16.000, SyringeSpeed=1000, SampleHeight=4.000

UdpMixWait Duration=2

UdpDraw From=ReagentBVial, Volume=0.000, SyringeSpeed=GlobalSpeed, SampleHeight=4.000

UdpInjectValve Position=Load

UdpDraw From=ReagentBVial, Volume=20.000, SyringeSpeed=1000, SampleHeight=4.000

UdpMixWait Duration=2

UdpInjectValve Position=Inject

UdpInjectMarker

UdpMixNeedleWash Volume=300.000

0.000 Autozero

NC_Pump.Flow = 0.300 [µl/min]

NC_Pump.%B = 99.0 [%]

Wait UV.Ready and LoadingPump.Ready and NC_Pump.Ready and ColumnOven.Ready and Sampler.Ready and PumpModule.Ready

Inject

LoadingPump_Pressure.AcqOn

NC_Pump_Pressure.AcqOn

NC_Pump_Press_RightBlk.AcqOn

NC_Pump_Press_LeftBlk.AcqOn

ColumnOven_Temp.AcqOn

UV_VIS_1.AcqOn

NC_Pump.Flow = 0.300 [µl/min]

NC_Pump.%B = 99.0 [%]

8.000 ValveLeft = 10_1

13.000 Start_MS.State On

13.100 Start_MS.State Off

40.000 NC_Pump.Flow = 0.300 [µl/min]

NC_Pump.%B = 99.0 [%]

41.000 NC_Pump.Flow = 0.300 [µl/min]

NC_Pump.%B = 3.0 [%]

45.000 ValveLeft = 1_2

60.000 NC_Pump.Flow = 0.300 [µl/min]

UV_VIS_1.AcqOff

LoadingPump_Pressure.AcqOff

NC_Pump_Pressure.AcqOff

NC_Pump_Press_RightBlk.AcqOff

NC_Pump_Press_LeftBlk.AcqOff

ColumnOven_Temp.AcqOff

NC_Pump.%B = 3.0 [%]

End
